# Publisher Correction: *UBE4B*, a *microRNA-9* target gene, promotes autophagy-mediated Tau degradation

**DOI:** 10.1038/s41467-021-24572-0

**Published:** 2021-07-07

**Authors:** Manivannan Subramanian, Seung Jae Hyeon, Tanuza Das, Yoon Seok Suh, Yun Kyung Kim, Jeong-Soo Lee, Eun Joo Song, Hoon Ryu, Kweon Yu

**Affiliations:** 1 Metabolism and Neurophysiology Research Group, KRIBB, Daejeon, Korea; 2 Convergence Research Center of Dementia, KIST, Seoul, Korea; 3 Center for Neuroscience, Brain Science Institute, KIST, Seoul, Korea; 4 Biomedical Research Institute, KIST, Seoul, Korea; 5 Graduate School of Pharmaceutical Sciences and College of Pharmacy, Ewha Womans University, Seoul, Korea; 6 Department of Functional Genomics, UST, Daejeon, Korea

**Keywords:** miRNAs, Ubiquitin ligases, Cell death in the nervous system, Alzheimer's disease

Correction to: *Nature Communications* 10.1038/s41467-021-23597-9, published online 2 June 2021.

In the original version of this Article, Figs. 4a, b, d, and e were missing the black circles indicating which plasmids were used in the experiments. This error has now been fixed, and the PDF and HTML versions of the Article contain corrected Fig. 4.

