# Supplementary material for: UBE4B, a microRNA-9 target gene, promotes autophagy-mediated Tau degradation
Source: Nat Commun. 2021 Jun 2;12:3291. doi: 10.1038/s41467-021-23597-9 (PMC8172564; doi:10.1038/s41467-021-23597-9)
Supplement: Supplementary file 1 — Supplemenatry Information [file 41467_2021_23597_MOESM1_ESM.pdf]

UBE4B, a *microRNA-9* target gene, promotes autophagy-mediated Tau degradation

Manivannan Subramanian[1, 2*], Seung Jae Hyeon[3*], Tanuza Das[4], Yoon Seok Suh[1],

Yun Kyung Kim[2], Jeong-Soo Lee[1, 2], Eun Joo Song[5†], Hoon Ryu[3†], Kweon Yu[1, 2, 6†]

[1] Metabolism and Neurophysiology Research Group, KRIBB, Daejeon 34141, Korea

[2] Convergence Research Center of Dementia, KIST, Seoul 02792, Korea

[3] Center for Neuroscience, Brain Science Institute, KIST, Seoul 02792, Korea

[4] Biomedical Research Institute, KIST, Seoul 02792, Korea

[5]Graduate School of Pharmaceutical Sciences and College of Pharmacy, Ewha Womans University, Seoul 03760, Korea

[6] Department of Functional Genomics, UST, Daejeon, Korea

*These authors contributed equally.

†Correspondence: kweonyu@kribb.re.kr, hoonryu@kist.re.kr, esong@ewha.ac.kr

Supplementary Figure 1: Eye sizes of *Drosophila* miRNA library from screening in *GMR>hTau*.

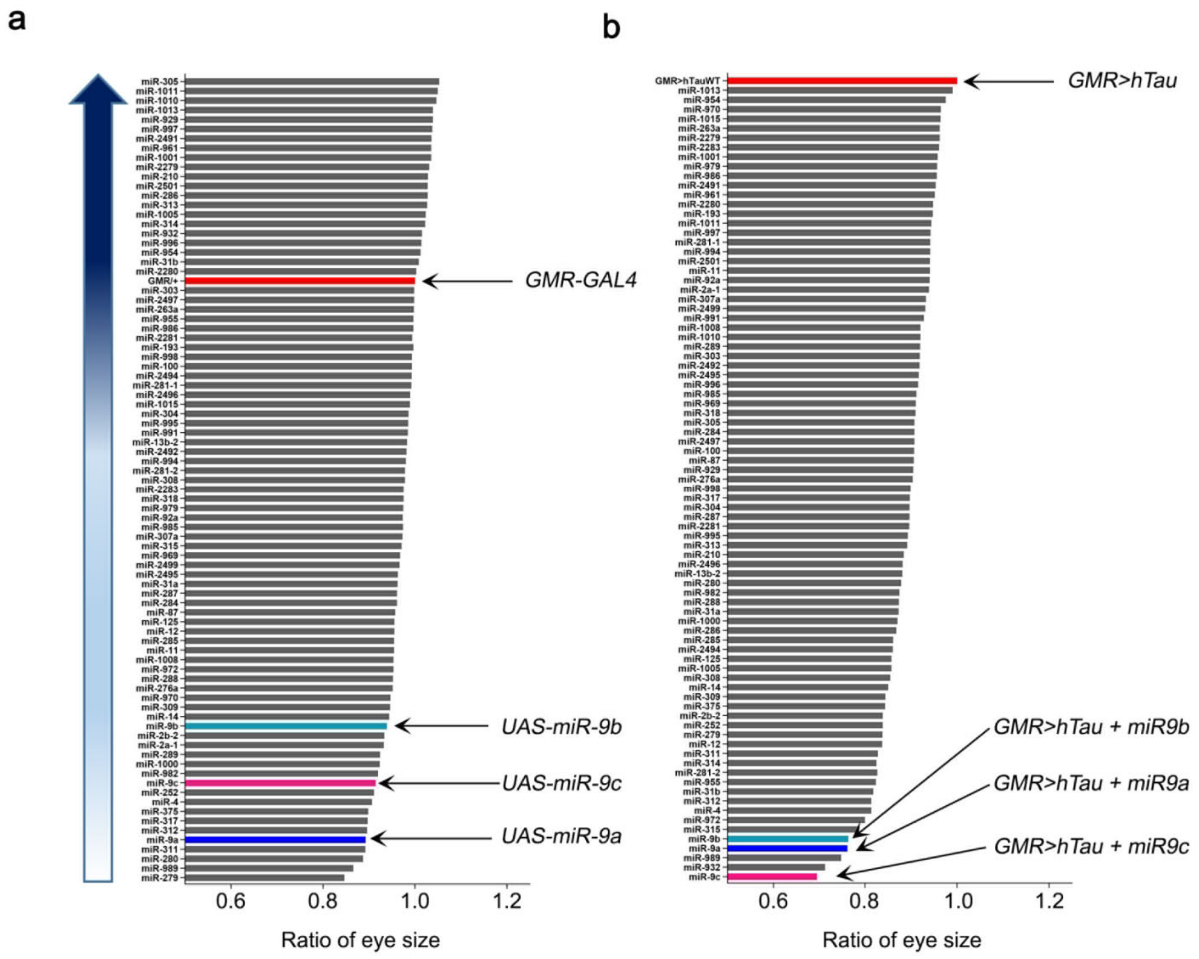

(a) Eye sizes of *Drosophila miRNA* library and *GMR-GAL4*. **(b)** Quantitative representation of Fig. 1A and B in order of increasing eye size. Colored bars indicate *miR-9* family miRNAs – *UAS-miR-9a*, *UAS-miR-9b*, *UAS-miR-9c* and are compared to their controls colored in red *GMR-GAL4/+* and *GMR>hTau* respectively.

Supplementary Figure 2: *Drosophila* eye sizes of *miR9a* target RNAi from screening in *GMR>hTau*.

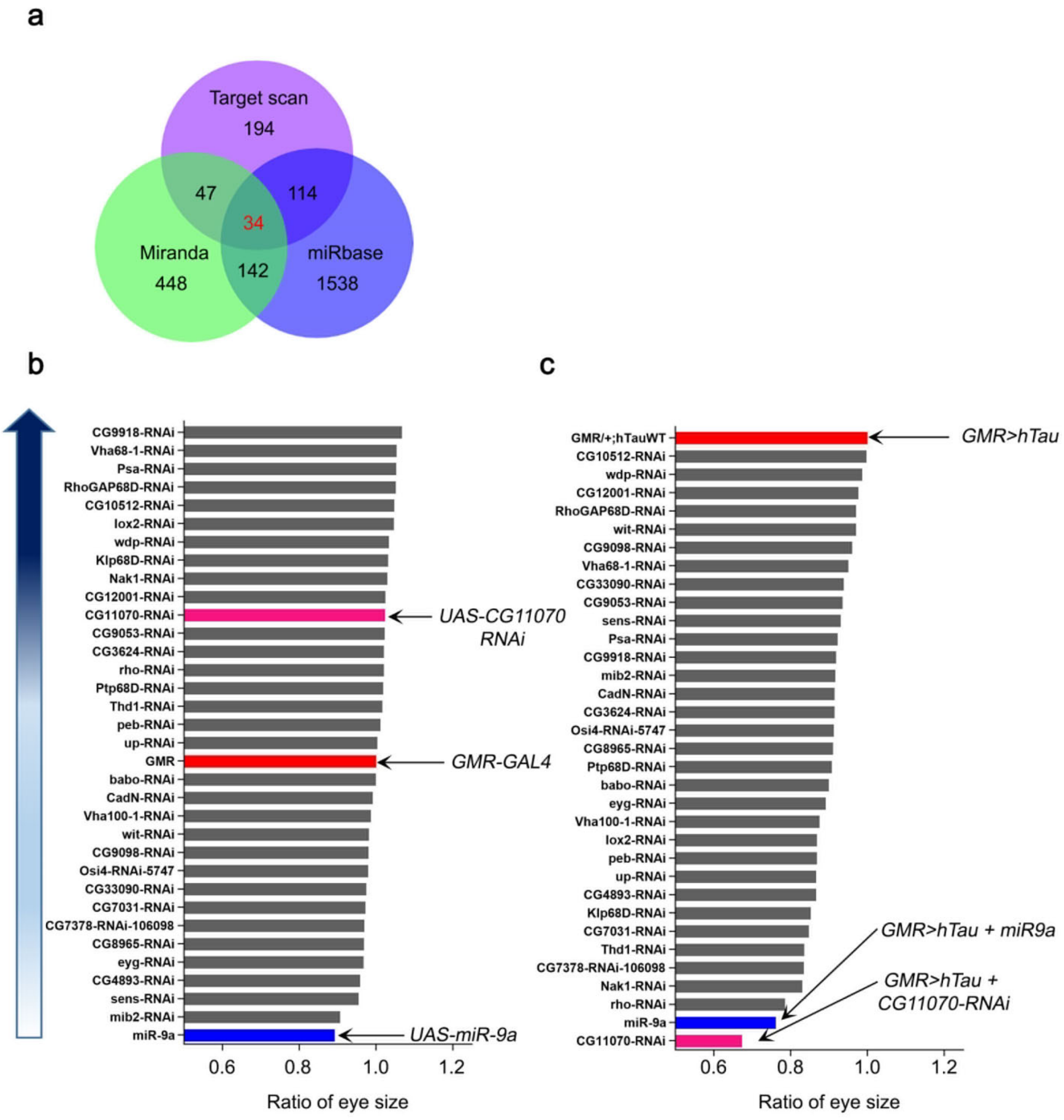

(a) Three different programs for microRNA target prediction (Targetscan, miR-base, and Miranda) identified 34 common *miR-9a* targets. **(b)** Eye sizes of *miR9a* target RNAi and *GMR-GAL4*. **(c)** Quantitative representation of Fig. 2A and B in order of increasing eye sizes. Colored bars indicate *UAS-miR-9a* and its target *UAS-CG11070-RNAi* and are compared to their controls colored in red *GMR-GAL4/+* and *GMR>hTau* respectively.

Supplementary Figure 3: Homology between *Drosophila CG11070* and human *UBE4B,* and western blotting with *CG11070* or *UBE4B* overexpression in *Drosophila*.

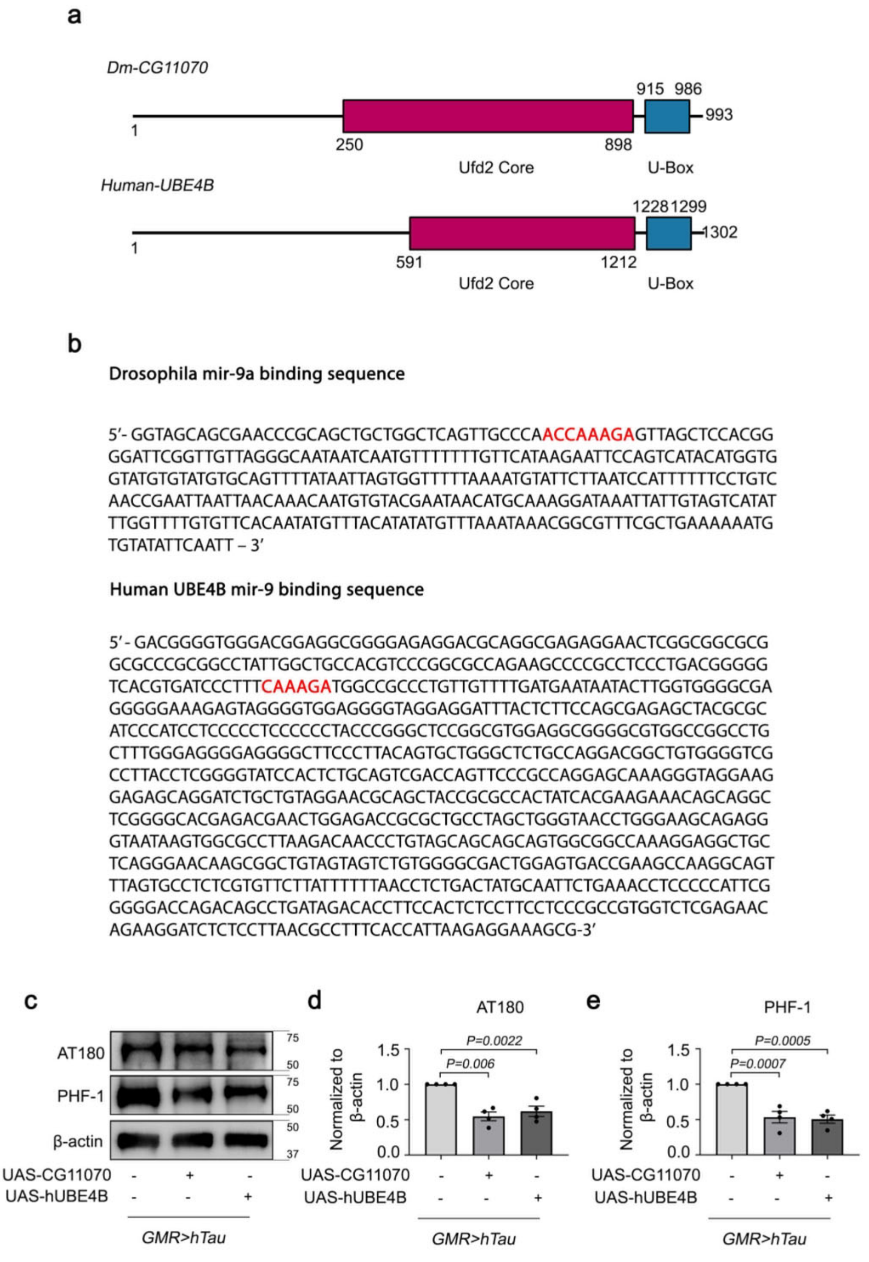

(a) Homologous domains between *Drosophila* CG11079 and human UBE4B proteins. **(b)** 3' UTR sequences of *Drosophila CG11070* and human *UBE4B* revealed that the 3' UTRs of each ortholog contained miR-9a/miR-9 binding sequences (red color). **(c-e)** Western

blotting revealed that the overexpression of *Drosophila CG11070* or mammalian *UBE4B* in *GMR>hTau* flies decreased phosphorylated Tau, as detected by AT180 (p-T231) and PHF-1 (p-S396/S404) antibodies, relative to the control *GMR>hTau* flies. N=4 biologically independent experiments. Data are presented as the mean ± s.e.m. Statistical significance was determined with two-tailed Student's *t*-test. Statistical source data

Supplementary Figure 4: The knockdown of *miR-9a* alleviated *hTau* phenotypes in *Drosophila*.

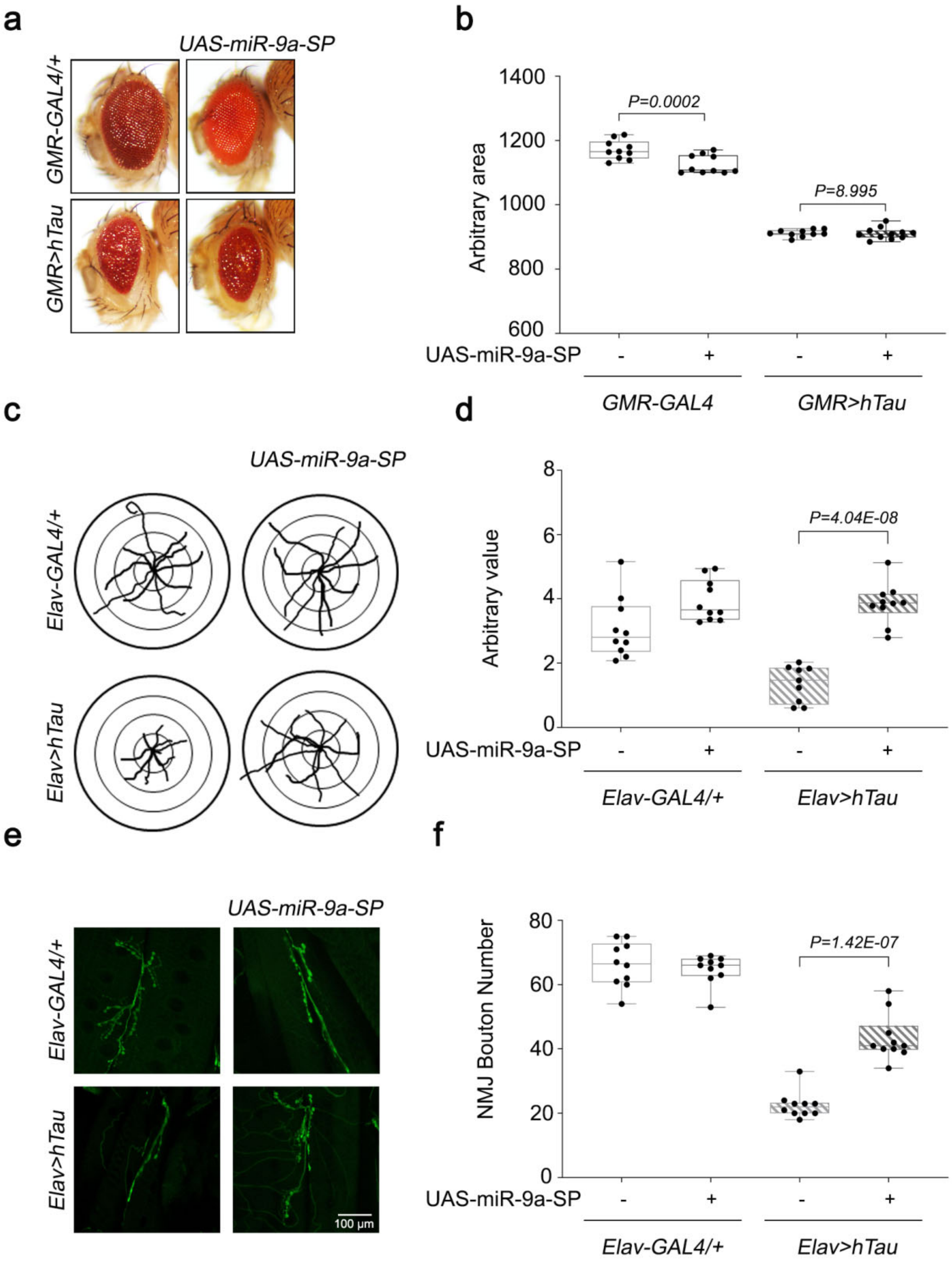

(a, b) Eye-specific knockdown of *Drosophila miR-9a* using *miR-9a-SP* unchanged eye size relative to *GMR>hTau* controls. N=10 biologically independent experiments. In the box plots the whiskers represent the 5th to 95th percentile range. **(c, d)** Neuronal knockdown of *Drosophila miR-9a* using *miR-9a-SP* using *Elav-Gal4* in *Elav>hTau* flies significantly increased larval crawling. N=10 biologically independent experiments. In the box plots the whiskers represent the 5th to 95th percentile range. **(e, f)** Neuronal knockdown of *Drosophila miR-9a* using *miR-9a-SP* using *Elav-Gal4* in *Elav>hTau* flies significantly increased synaptic bouton numbers relative to *Elav>hTau* controls. Scale bar 100µm. N=10 biologically independent experiments. In the box plots the whiskers represent the 5th to 95th percentile range. Data are presented as the mean ± s.e.m. Statistical significance was determined with two-tailed Student's *t*-test. Statistical source data

Supplementary Figure 5: The knockdown of *STUB1* affects Tau degradation in neuroblastoma cells.

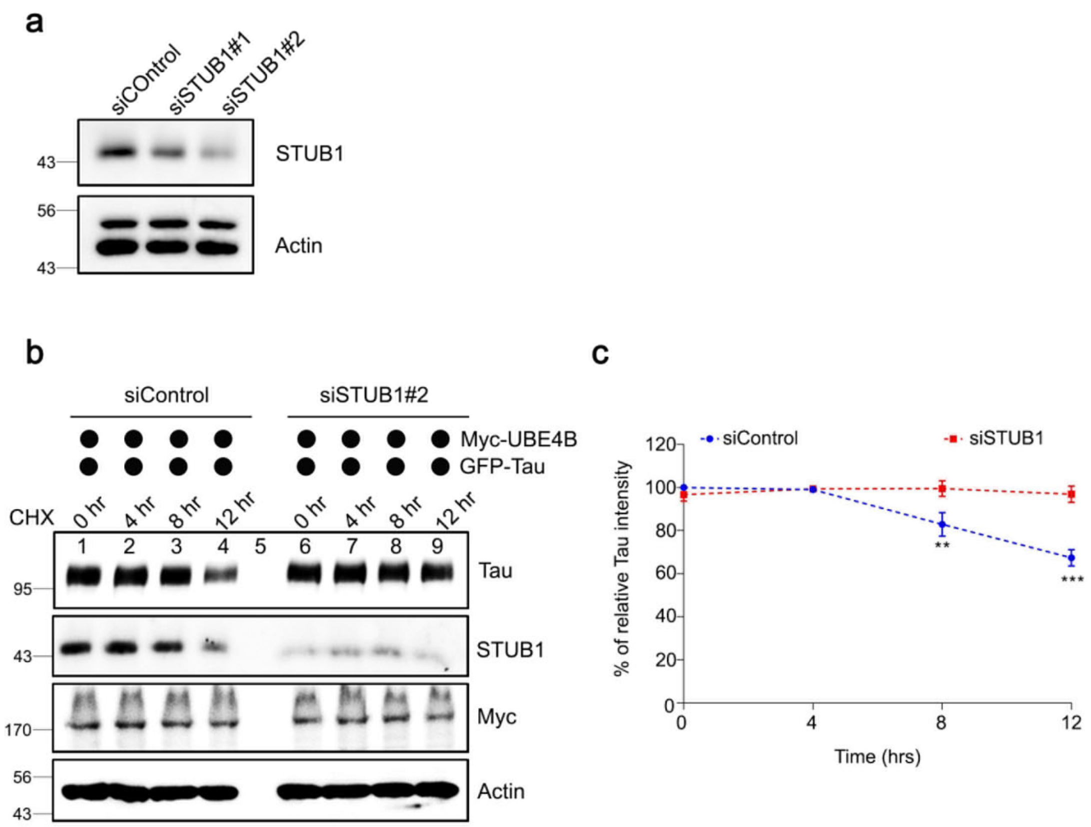

(a) The knockdown of *STUB1* with two different siRNAs in SH-SY5Y cells showed decreased STUB1 protein levels. **(b)** Tau degradation was inhibited by *siSTUB1*. All western blots were performed three times. (**c**) Quantification of Tau degradation in *siSTUB1* expressing cells. N=3 biologically independent experiments. Data are presented as the mean ± s.e.m. Statistical significance was determined with two-tailed Student's *t*-test. Statistical source data.

Supplementary Figure 6: UBE4B and STUB1 decrease Tau phosphorylation in the Tau-BiFC mouse model.

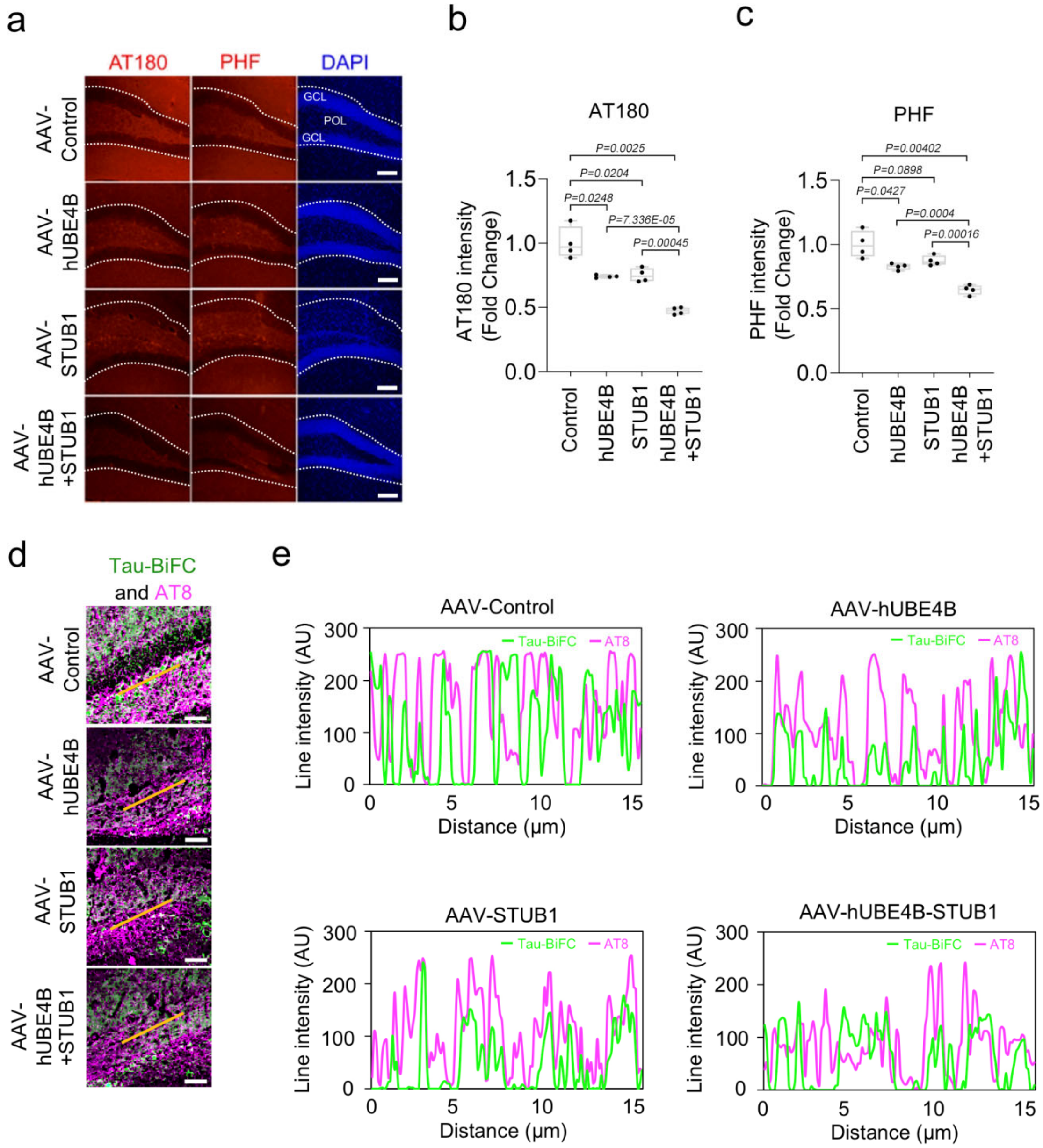

(a) *AAV-UBE4B* and *AAV-STUB1* decreased pTau levels AT180 (p-T231) and PHF-1 (pS396/S404) in the dentate gyrus compared to AAV-Control. Scale bars (white), 40μm. These experiments were performed four times. **(b, c)** Densitometry analysis revealed that *AAV-UBE4B* and *STUB1* significantly decreased pTau levels (AT180 and PHF-1) in the dentate gyrus relative to *AAV-Control* (*AAV-Control* N=4; *AAV-UBE4B* N=4; *AAV-STUB1* N=4; *AAV-UBE4B+AAV-STUB1* N=4; N=4 biologically independent animals). Co-delivery

of *AAV-UBE4B* and *AAV-STUB1* showed an additive reduction of pTau levels (AT180 and PHF-1) in the dentate gyrus compared to *AAV-UBE4B* or *AAV-STUB1* only. In the box plots the whiskers represent the 5th to 95th percentile range. Statistical significance was determined with two-tailed Student's *t*-test. Statistical source data. (**d)** Colocalization analysis (orange line) shows that Tau-BiFC (green) and AT8 (S202/T205) (purple) levels are correlatively reduced in the polymorphic layer (POL) of dentate gyrus through *AAV-UBE4B* and *AAV-STUB1*-dependent manner*.* The image data was originated from the main Figure 6e. Scale bars (white), 20μm. **(e)** Intensity of Tau-BiFC and AT8 (S202/T205) levels are correlatively decreased through *AAV-UBE4B* and *AAV-STUB1*-dependent manner compared to *AAV-Control.* Data are presented as means ± s.e.m. Statistical significance was determined with two-tailed Student's *t*-test. Statistical source data

Supplementary Figure 7: Autophagy inhibitors increase pTau accumulation in the Tau-BiFC mouse model.

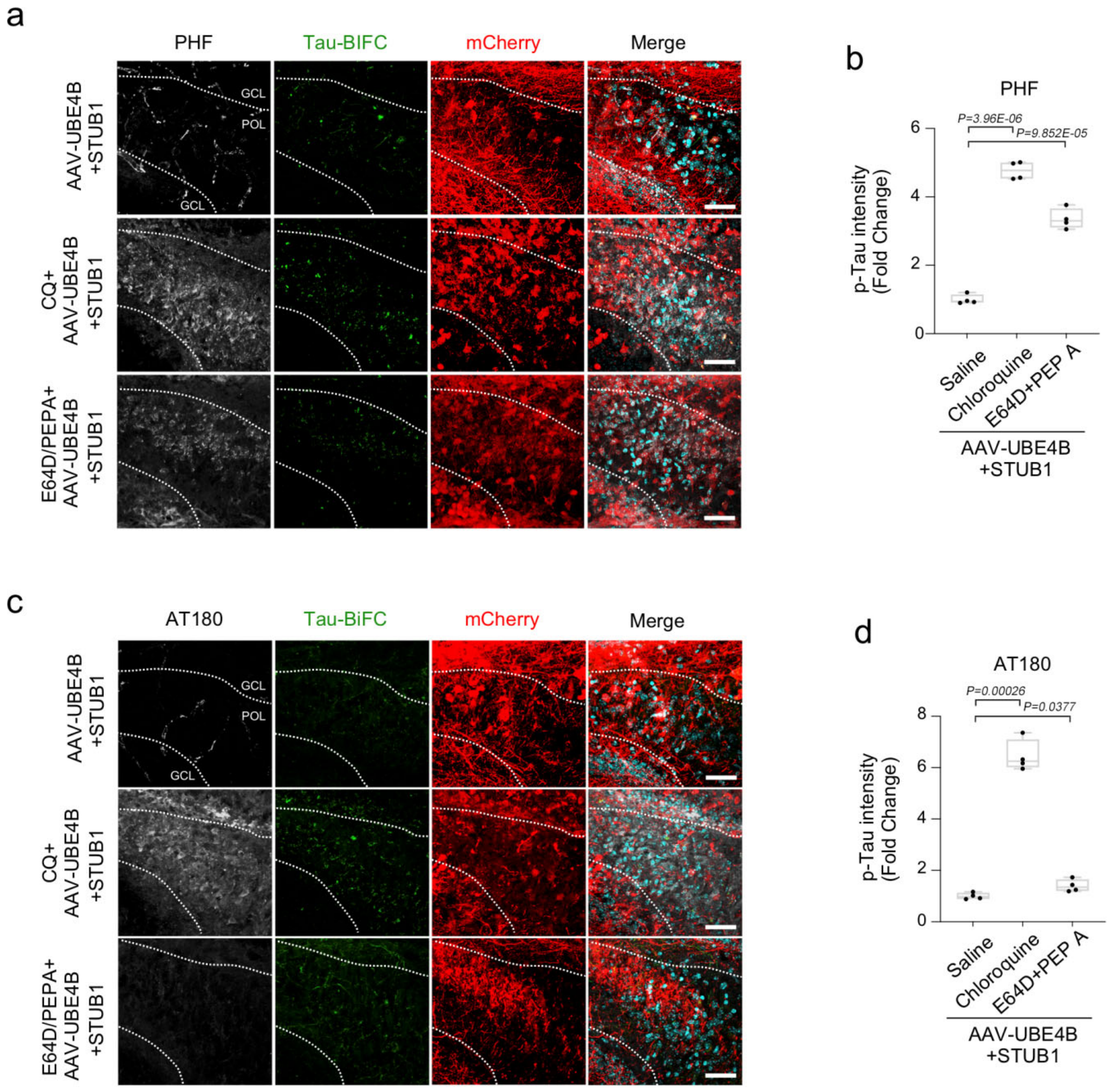

(a) Chloroquine (CQ) and E64D plus PEPA treatment increased pTau (PHF-1:S396/S404) levels in the dentate gyrus relative to the control. GCL, granular cell layer; POL, polymorphic layer. Scale bars (white), 40μm. These experiments were performed four times. **(b)** Densitometry analysis revealed that autophagy inhibitors significantly increased pTau (PHF-1:S396/S404) levels in dentate gyrus relative to the controls (*AAV-UBE4B+AAV-STUB1* (saline control), N=4; CQ+*AAV-UBE4B+AAV-STUB1*, N=4; E64D/PEPA+*AAV-UBE4B+AAV-STUB1*, N=4; N=4 biologically independent animals). In the box plots the whiskers represent the 5th to 95th percentile range. **(c)** CQ and

E64D+PEPA treatment increased pTau (AT180: T231) levels in the dentate gyrus relative to the control. Scale bars (white), 40μm. These experiments were performed four times. **(d)** Densitometry analysis revealed that autophagy inhibitors significantly increased pTau (AT180: T231) in the dentate gyrus relative to the controls (*AAV-UBE4B+AAV-STUB1* (saline control), N=4; CQ+*AAV-UBE4B+AAV-STUB1*, N=4; E64D/PEPA+*AAV-UBE4B+AAV-STUB1*, N=4; N=4 biologically independent animals). In the box plots the whiskers represent the 5th to 95th percentile range. Data are presented as means ± s.e.m. Statistical significance was determined with two-tailed Student's *t*-test. Statistical source data.

Supplementary Figure 8: Autophagy inhibitors affects beclin (BECN1) accumulation in the TauBiFC mouse model.

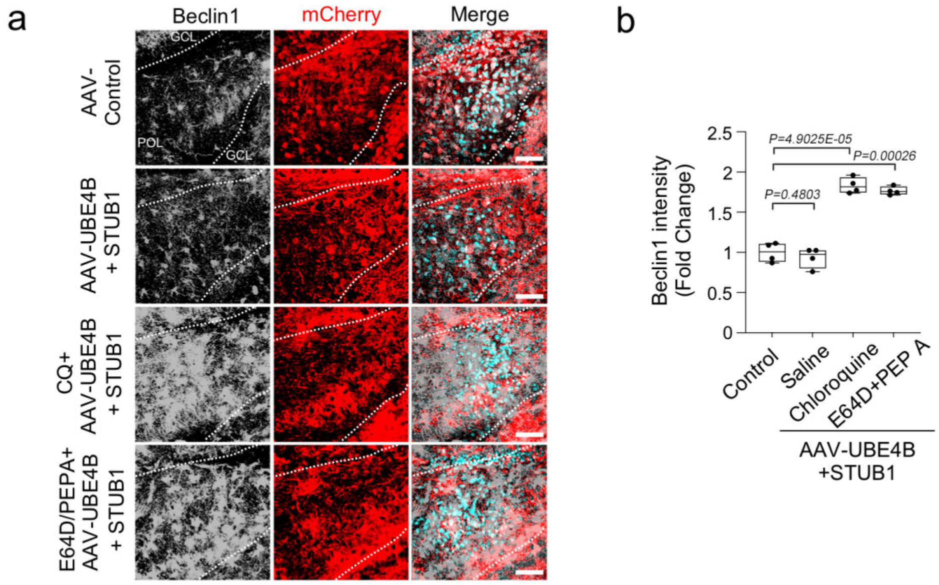

(a) Chloroquine (CQ) and E64D plus PEPA treatment increased BECN1 levels in the dentate gyrus relative to the control. Scale bars (white), 40μm. These experiments were performed four times. **(b)** Densitometry analysis revealed that autophagy inhibitors significantly increased BECN1 levels in the dentate gyrus relative to the controls (*AAV-UBE4B+AAV-STUB1 (saline control)*, N=4; CQ+*AAV-UBE4B+AAV-STUB1*, N=4; E64D/PEPA+*AAV-UBE4B+AAV-STUB1*, N=4; N=4 biologically independent animals). In the box plots the whiskers represent the 5th to 95th percentile range. Data are presented as means ± s.e.m. Statistical significance was determined with two-tailed Student's *t*-test. Statistical source data.

Supplementary Table 1. PCR primers used for miRNA–mRNA pull-down assay

PCR primer	Primer sequence (5' to 3')
RP49	F: AGGGTATCGACAACAGAGTG<br>R: CACCAGGAACTTCTTGAATC
CG11070	F: GTAGCAGCGAACCCGCAG<br>R: AATTCGGTTGACAGGAAAAA
senseless	F: TGGCAGCTAAACGTACCAAA<br>R: GATCGTATAAATAAATGTGG
sNPFR1	F: GGGCCATTTCGCATATTTAC<br>R: ATTTAATTCCGTGCGACTGG
tubulin	F: ACTGCAGCATCCTGTGAACC<br>R: TGGGAACATTTCCGTTTGAT
