# Supplementary material for: UBE4B, a microRNA-9 target gene, promotes autophagy-mediated Tau degradation
Source: Nat Commun. 2021 Jun 2;12:3291. doi: 10.1038/s41467-021-23597-9 (PMC8172564; doi:10.1038/s41467-021-23597-9)
Supplement: Supplementary file 3 — Reporting Summary [file 41467_2021_23597_MOESM3_ESM.pdf]

nature research

Corresponding author(s): Kweon Yu

Last updated by author(s): Apr 22, 2021

# Reporting Summary

Nature Research wishes to improve the reproducibility of the work that we publish. This form provides structure for consistency and transparency in reporting. For further information on Nature Research policies, see our Editorial Policies and the Editorial Policy Checklist.

## Statistics

For all statistical analyses, confirm that the following items are present in the figure legend, table legend, main text, or Methods section.

| n/a | Confirmed | |
|---|---|---|
| ☐ | ☒ | The exact sample size ($n$) for each experimental group/condition, given as a discrete number and unit of measurement |
| ☐ | ☒ | A statement on whether measurements were taken from distinct samples or whether the same sample was measured repeatedly |
| ☐ | ☒ | The statistical test(s) used AND whether they are one- or two-sided<br>*Only common tests should be described solely by name; describe more complex techniques in the Methods section.* |
| ☐ | ☒ | A description of all covariates tested |
| ☐ | ☒ | A description of any assumptions or corrections, such as tests of normality and adjustment for multiple comparisons |
| ☐ | ☒ | A full description of the statistical parameters including central tendency (e.g. means) or other basic estimates (e.g. regression coefficient) AND variation (e.g. standard deviation) or associated estimates of uncertainty (e.g. confidence intervals) |
| ☐ | ☒ | For null hypothesis testing, the test statistic (e.g. $F$, $t$, $r$) with confidence intervals, effect sizes, degrees of freedom and $P$ value noted<br>*Give P values as exact values whenever suitable.* |
| ☒ | ☐ | For Bayesian analysis, information on the choice of priors and Markov chain Monte Carlo settings |
| ☒ | ☐ | For hierarchical and complex designs, identification of the appropriate level for tests and full reporting of outcomes |
| ☒ | ☐ | Estimates of effect sizes (e.g. Cohen's $d$, Pearson's $r$), indicating how they were calculated |

*Our web collection on statistics for biologists contains articles on many of the points above.*

## Software and code

Policy information about availability of computer code

| | |
|---|---|
| Data collection | Data collection was done using GraphPad Prism Software 9.1.0 (GraphPad Software, CA, USA) and Image J v1.44 software ((National Institutes of Health, Bethesda, USA). |
| Data analysis | All experiments were performed more than three times. In case of animal study, 'N' represents the number of biologically independent animals. Boxplots were generated using the standard style except that the whiskers represent minimum to maximum. In bar charts, unless otherwise noted, data are presented as mean ± SEM, and comparisons between groups were conducted using the Student's t-test considering p<0.05 to be statistically significant. For multiple comparisons, we performed a one-way analysis of variance followed by pairwise t-tests using the Bonferroni method to adjust the p-value threshold for significance. |

For manuscripts utilizing custom algorithms or software that are central to the research but not yet described in published literature, software must be made available to editors and reviewers. We strongly encourage code deposition in a community repository (e.g. GitHub). See the Nature Research guidelines for submitting code & software for further information.

## Data

Policy information about availability of data

All manuscripts must include a data availability statement. This statement should provide the following information, where applicable:

- Accession codes, unique identifiers, or web links for publicly available datasets
- A list of figures that have associated raw data
- A description of any restrictions on data availability

The data that support the findings of this study are available from the corresponding author upon reasonable request. Source data are provided with this paper.

# Field-specific reporting

Please select the one below that is the best fit for your research. If you are not sure, read the appropriate sections before making your selection.

☒ Life sciences ☐ Behavioural & social sciences ☐ Ecological, evolutionary & environmental sciences

For a reference copy of the document with all sections, see nature.com/documents/nr-reporting-summary-flat.pdf

# Life sciences study design

All studies must disclose on these points even when the disclosure is negative.

| | |
|---|---|
| Sample size | Sample sizes of fly and mouse experiments were empirically determined. Progressively increasing numbers of mice from experiments to reach statistical significance between groups. |
| Data exclusions | There are no data exclusions of fly and mice experiments in this manuscript. |
| Replication | All fly experiments were repeated at least three times and all mice experiments were repeated at least four times. |
| Randomization | Experimental and control animals bred in identical conditions were randomized in each experiment. All animals were randomly assigned into the tested group after manipulation or treatment. |
| Blinding | No blinding was done in fly and mouse experiments. All experiments were performed with multiple steps including the material preparation, treatment, biochemical and molecular analysis, as well as behavioral assay. Fly and mouse experiments were not amenable to blinding as subject from control and test group conditions become evident due to obvious differences in data obtained from downstream analyses. Furthermore, all experimental data for fly and mouse studies were generated by quantitative method of analyses that are not affected by user bias. |

# Reporting for specific materials, systems and methods

We require information from authors about some types of materials, experimental systems and methods used in many studies. Here, indicate whether each material, system or method listed is relevant to your study. If you are not sure if a list item applies to your research, read the appropriate section before selecting a response.

## Materials & experimental systems

| n/a | Involved in the study | |
|---|---|---|
| ☐ | ☒ | Antibodies |
| ☐ | ☒ | Eukaryotic cell lines |
| ☒ | ☐ | Palaeontology and archaeology |
| ☐ | ☒ | Animals and other organisms |
| ☒ | ☐ | Human research participants |
| ☒ | ☐ | Clinical data |
| ☒ | ☐ | Dual use research of concern |

## Methods

| n/a | Involved in the study | |
|---|---|---|
| ☒ | ☐ | ChIP-seq |
| ☒ | ☐ | Flow cytometry |
| ☒ | ☐ | MRI-based neuroimaging |

## Antibodies

| | |
|---|---|
| Antibodies used | Antibodies for western blotting - Anti-Tau (1:1,000, T46, Cat no. 13-6400, Invitrogen), anti-AT180 (1:1,000, Cat no. MN1040, Invitrogen), anti-PHF-1 (1:1,000, Cat no. MN1050, Invitrogen), anti-AT8 (1:1,000, Cat no. MN1020, Invitrogen), anti-β-actin (1:1,000, Cat no. JLA20, DHSB), anti-Tau (Cat no. ab64193, Abcam), anti-β-actin (Cat no. LF-PA0207, AB Frontier), anti-Myc (Cat no. C3956, Sigma), anti-HA (Cat no. H6908, Sigma) and anti-CHIP (Cat no. sc-133066, Santa Cruz Biotechnology).<br>Antibodies for immunofluorescence - anti-Tau 5 (1:200, ab3931, Abcam), anti-pTau AT8 (1:200, Cat no. MN1020, Invitrogen), anti-pTau AT180 (1:200, Cat no. MN1040, Invitrogen), anti-pTau PHF-1 (1:200, Cat no. MN1050, Invitrogen),anti-LC3 (1:200, Cat no. M152-3, MBL), anti-P62 (1:200, Cat no. PM045, MBL) and anti-Beclin (1:200, Cat no. PD017, MBL). |
| Validation | All commercial antibody lots are routinely tested by the manufactures. Anti-Tau is validated by Invitrogen and is suitable for WB. Anti-AT8, anti-PHF-1 and anti-AT180 are validated by Invitrogen and are suitable for WB, IHC, IP. Anti-Tau5 is validated by abcam and is suitable for IHC. Anti-LC3, anti-P62 and anti-Beclin are validated by MBL and are suitable for IHC. |

## Eukaryotic cell lines

Policy information about cell lines

| | |
|---|---|
| Cell line source(s) | SHSY5Y neuroblastoma cells was obtained from American Type Culture Collection (ATCC) |

| | |
|---|---|
| Authentication | Cell line authenticity was verified using Short Tandem Repeat (STR) analysis. |
| Mycoplasma contamination | All cell lines were mycoplasma negative confirmed by standard PCR method |
| Commonly misidentified lines (See ICLAC register) | No commonly misidentified cell lines were used in this study |

## Animals and other organisms

Policy information about studies involving animals; ARRIVE guidelines recommended for reporting animal research

| | |
|---|---|
| Laboratory animals | Drosophila melanogaster were maintained at 25oC on standard cornmeal, yeast, sugar, and agar medium. UAS-hTau, GMR-GAL4, and ElavGAL4 fly lines were obtained from Bloomington Stock Centre (Bloomington, USA). miR-9a target RNAi stocks were obtained from Bloomington Stock Centre (Bloomington, USA) and Vienna Drosophila Research Centre (Vienna, Austria). pUAS-CG11070 and pUAS-UBE4B flies were generated by the p-element-mediated germline transformation method with cDNA containing the coding regions of CG11070 or UBE4B. Male flies were used for all the experiments. Male TauP301L-BiFC mice was used for all the mice experiments |
| Wild animals | This study did not involve wild type animals |
| Field-collected samples | This study did not involve field-collected samples. |
| Ethics oversight | All mice experiments were approved by the Institutional Animal Care and Use Committee of Korea Institute of Science and Technology. |

Note that full information on the approval of the study protocol must also be provided in the manuscript.
