# Supplementary figures and images for: UBE4B, a microRNA-9 target gene, promotes autophagy-mediated Tau degradation
Source: Nat Commun. 2021 Jun 2;12:3291. doi: 10.1038/s41467-021-23597-9 (PMC8172564; doi:10.1038/s41467-021-23597-9)

**Fig.3g**

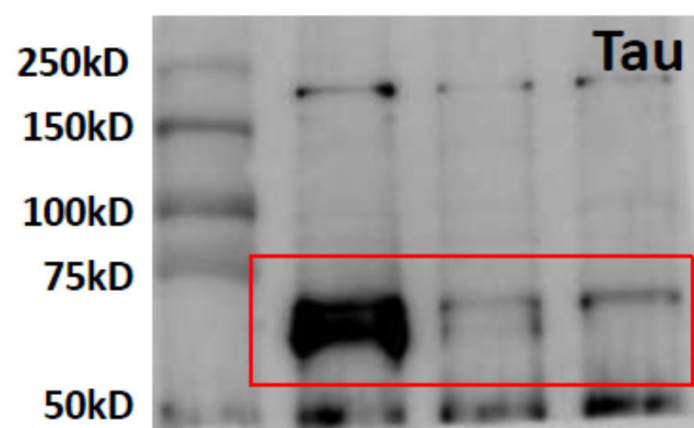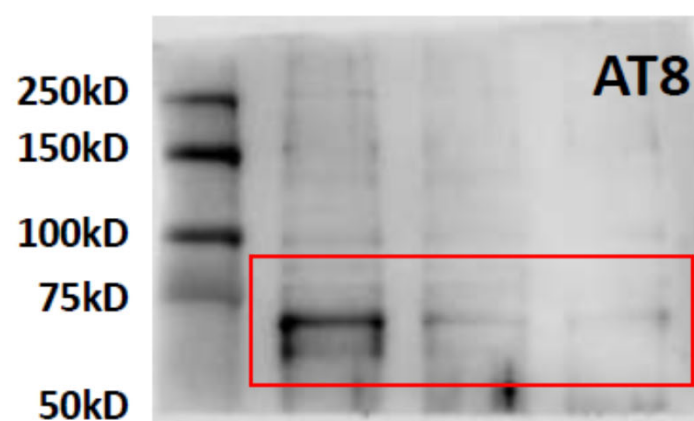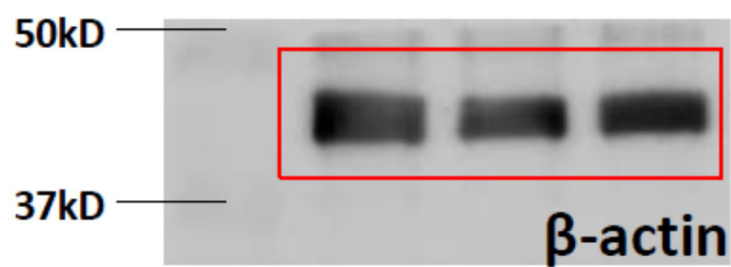

Fig.4a

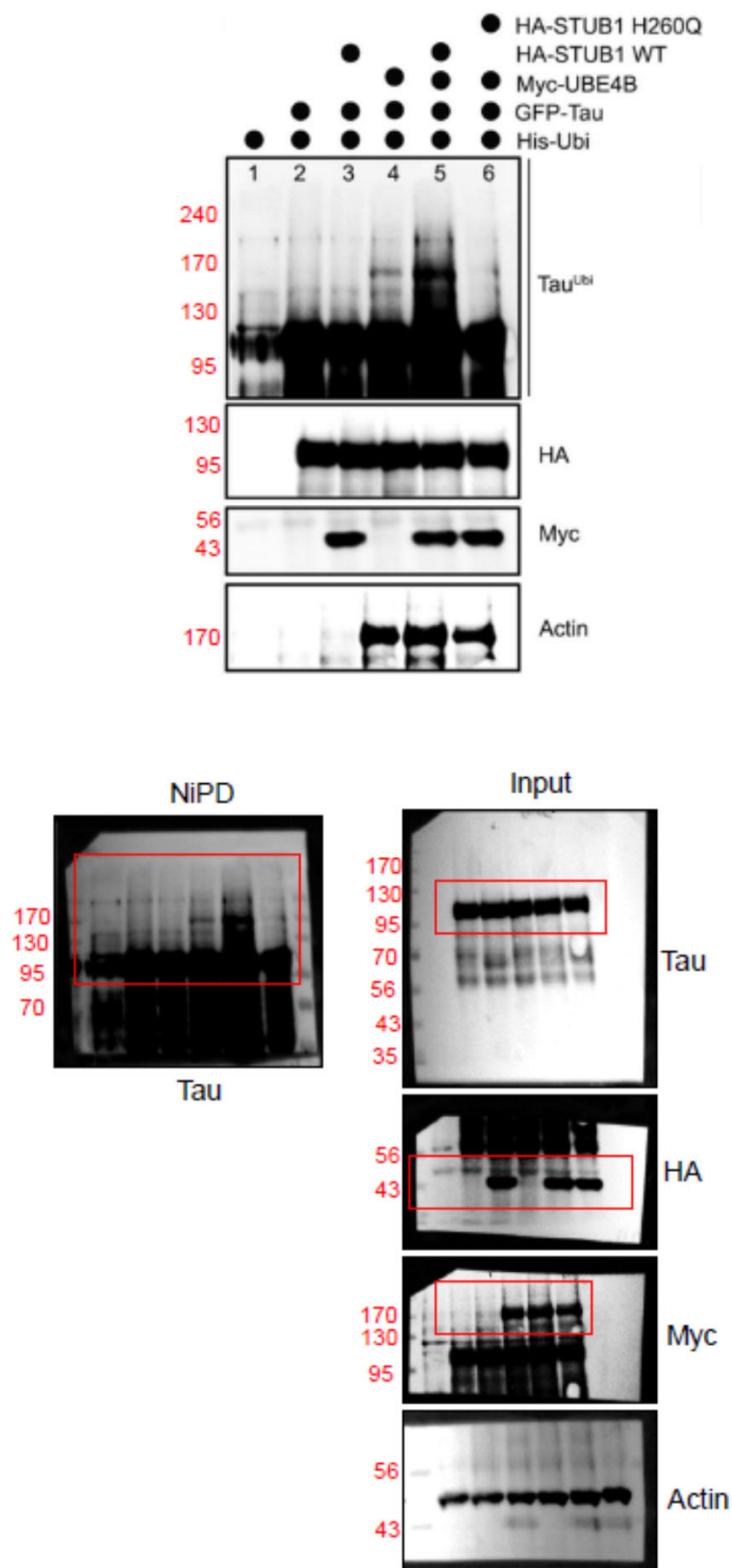

**Fig.4b**

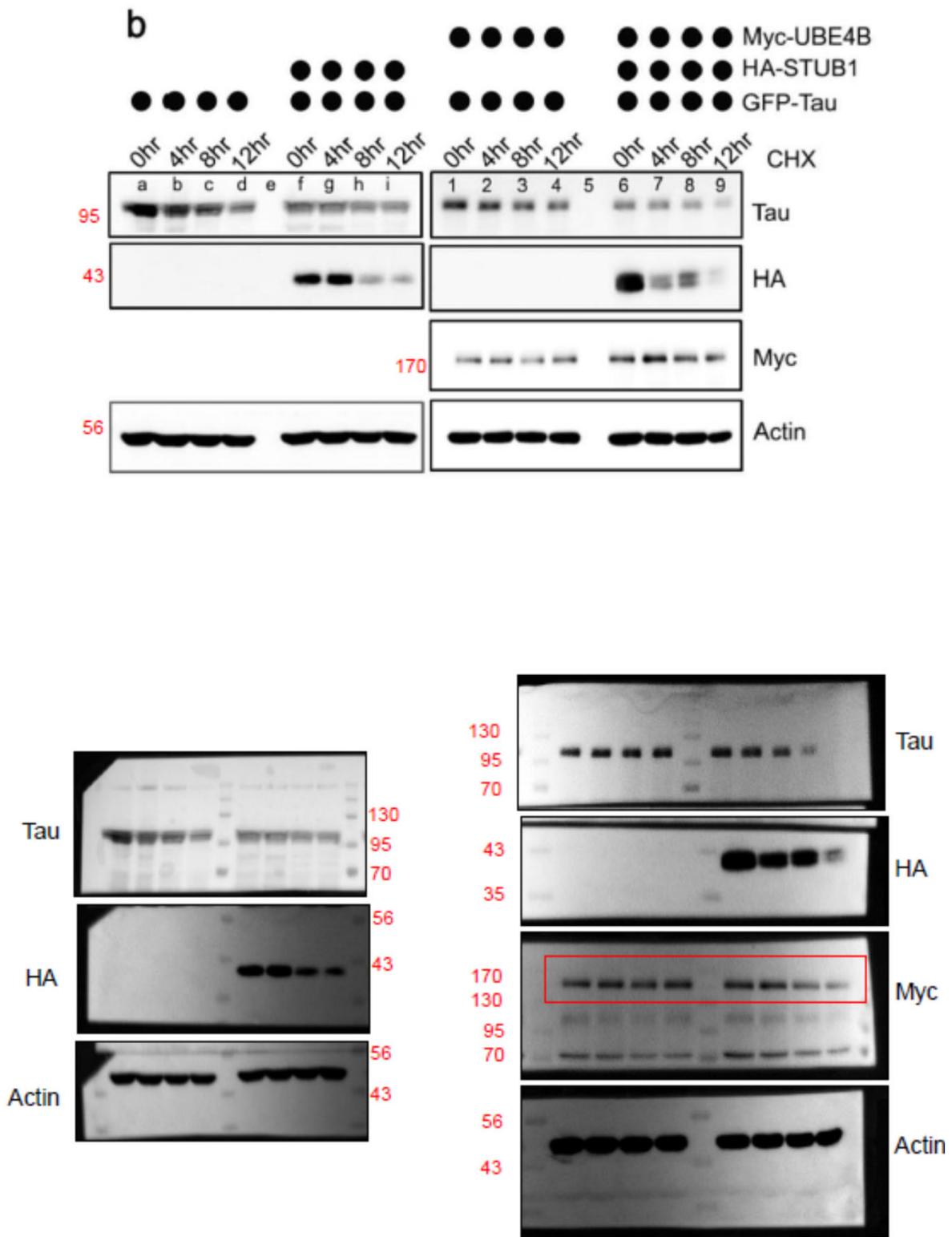

**Fig.4d**

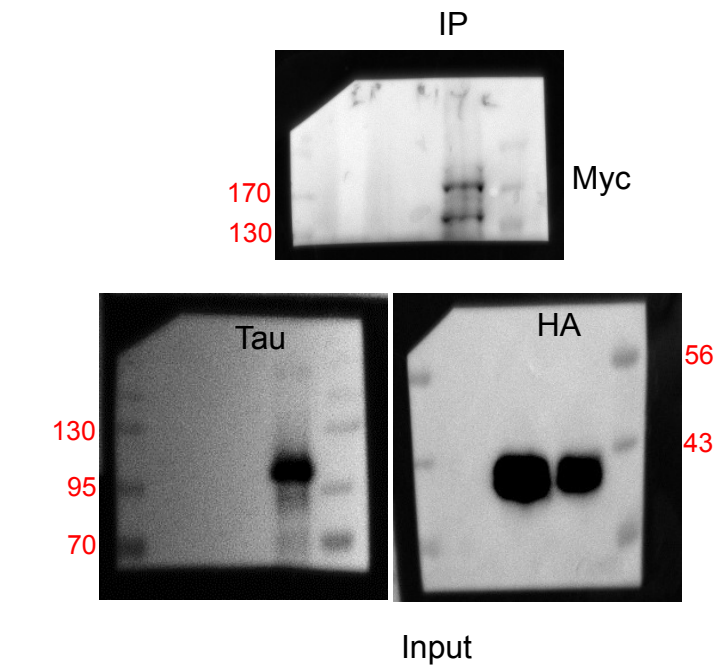

**Fig.4e**

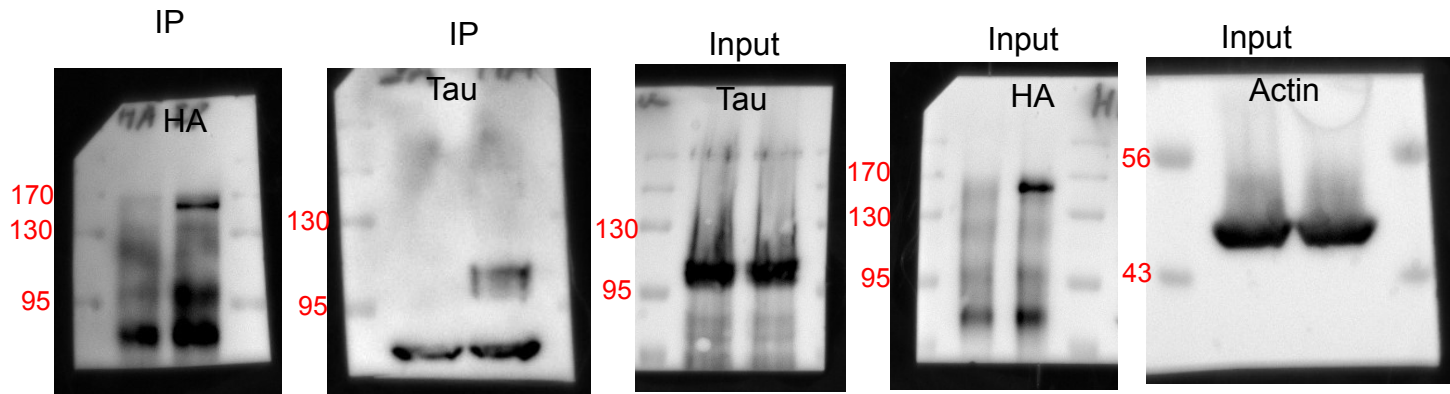

Fig.7a, b, c

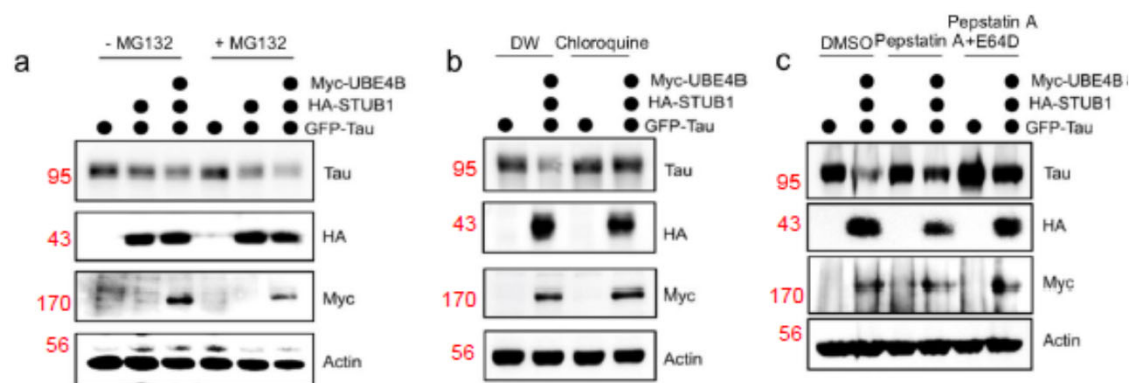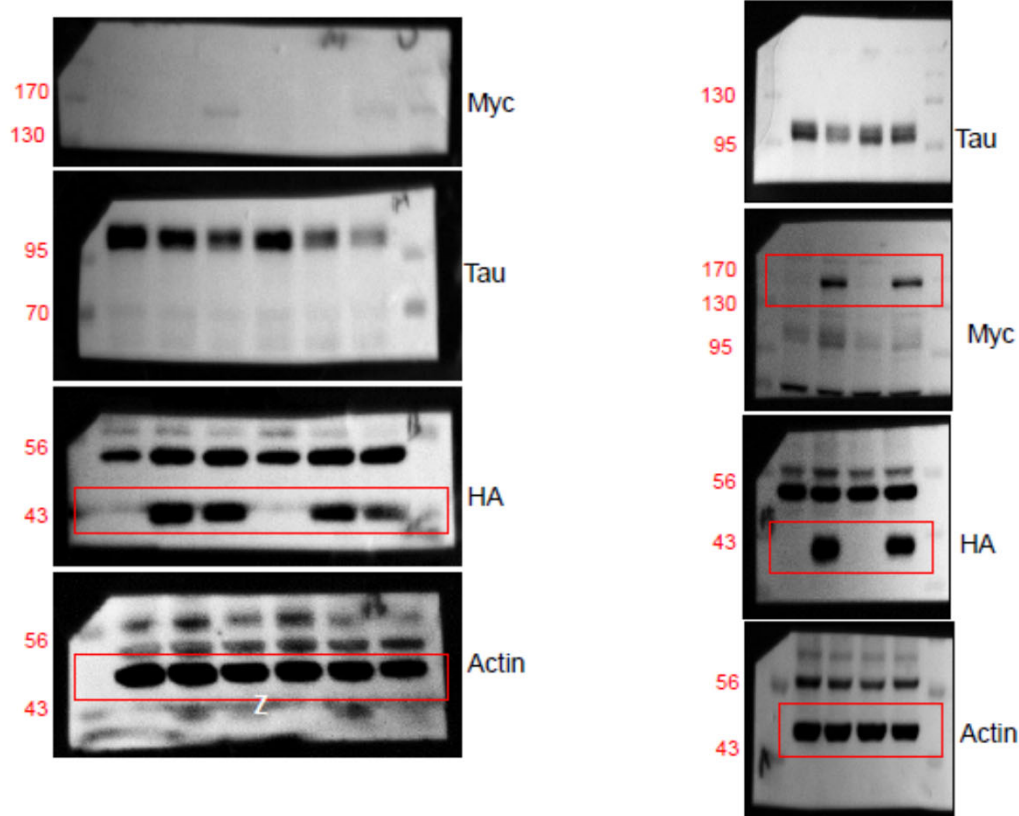

**S Fig.3c**

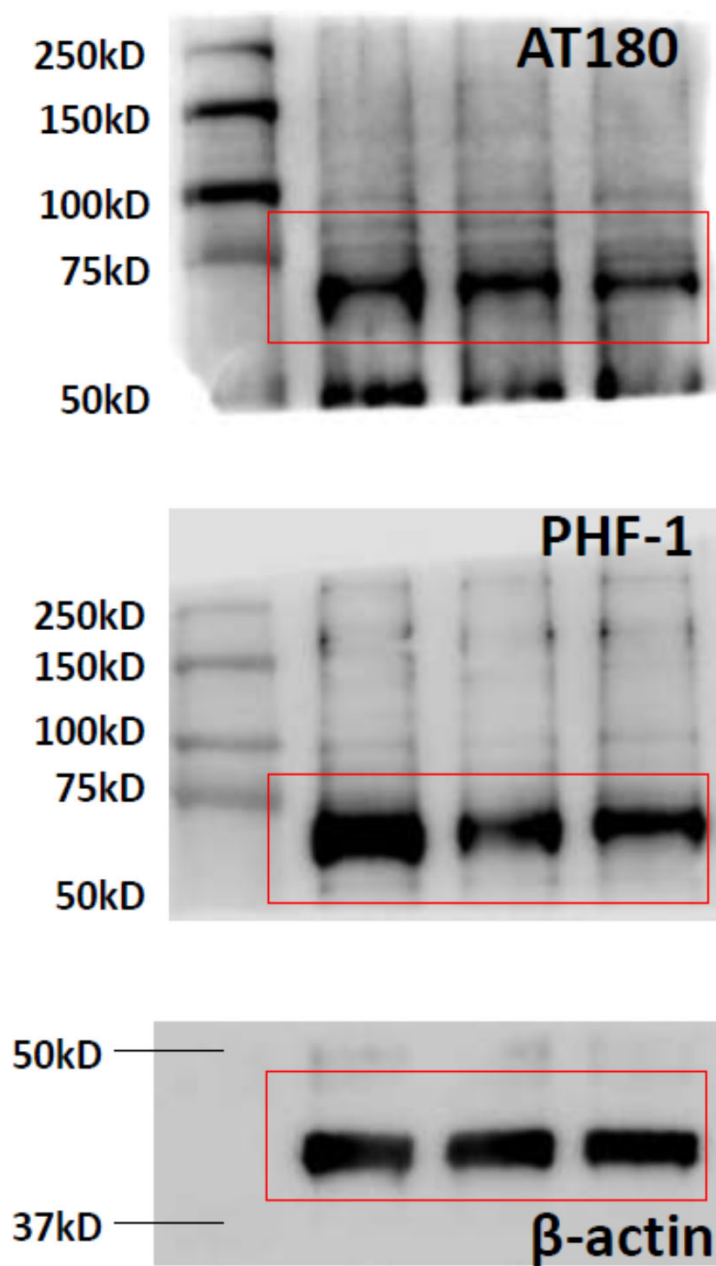

S Fig.5

S Fig.5

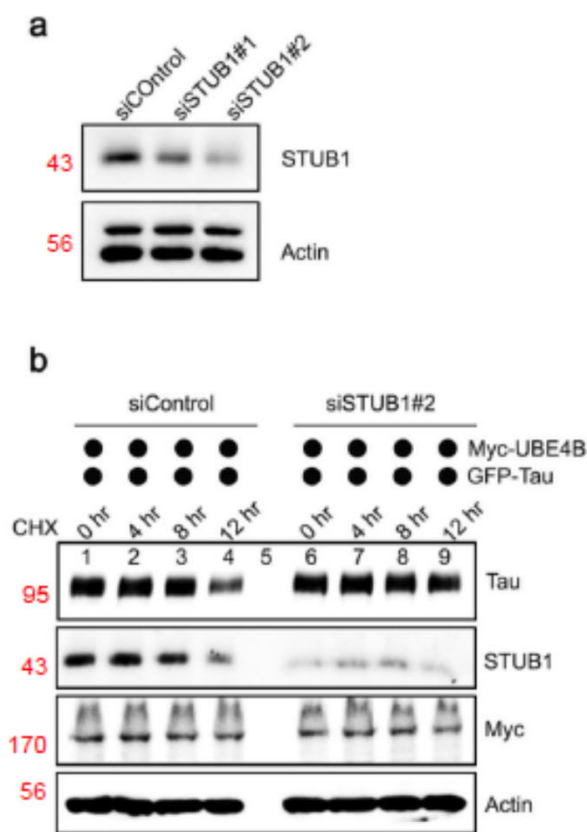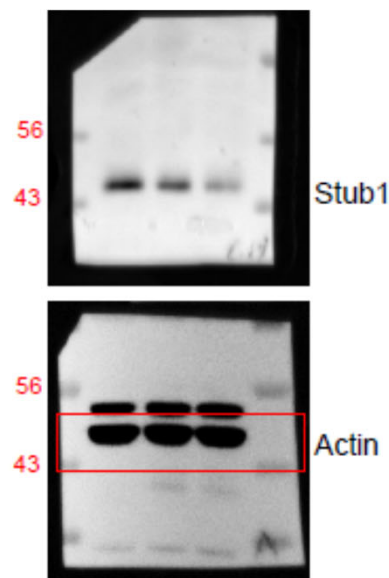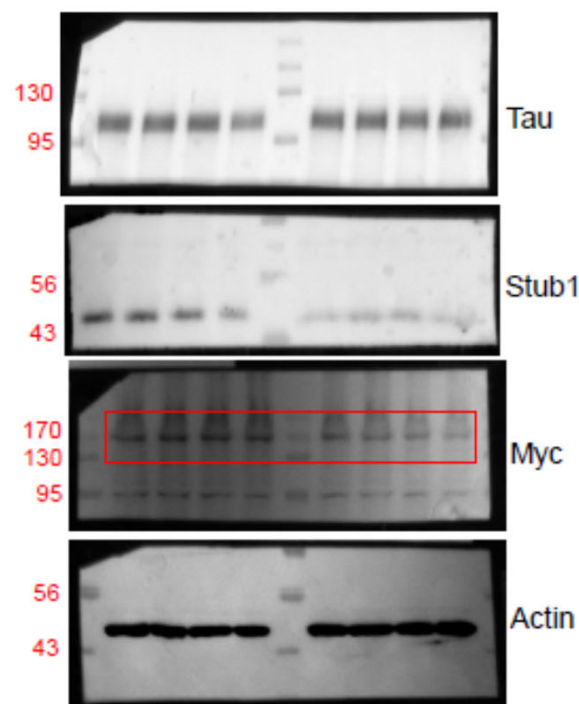

Supplement: Supplementary file 4 — Source data [file 41467_2021_23597_MOESM4_ESM.zip › Uncropped blots.pdf]
